# Osteosarcoma of mandible initially resembling lesion of dental periapex: a case report

**DOI:** 10.1016/S1808-8694(15)31318-5

**Published:** 2015-10-20

**Authors:** Rosilene C. Soares, Andréa F. Soares, Lélia B. Souza, Aldo L. V. dos Santos, Leão P. Pinto

**Affiliations:** 1 Ph.D. studies in Oral Pathology under course/UFRN; 2 Master studies in Oral Pathology under course/UFRN; 3 Professor, Ph.D. in Oral Pathology/UFRN; 4 Bucco-Maxillo-Facial Surgeon; 5 Professor, Ph.D. in Oral Pathology/UFRN Post-graduation Program in Oral Pathology – Federal University of Rio Grande do Norte (UFRN)

**Keywords:** osteossarcoma, mandibule, malignant neoplasm of the jaw bones

## Abstract

Osteosarcoma is a malignant mesenchymal tumor whose cancerous cells produce osteoid matrix. It is the most common primary malignant bone tumor, accounting for approximately 20% of the sarcomas, but only 5% of the osteosarcomas occur in the jaws. They present various clinical and histological aspects, as well as variable disease progression and outcome. This article shows a case report of a 20-year-old woman who presented swelling near the mandibular left premolar. After clinical diagnosis of lesion of the dental periapex, the patient initially underwent endodontic treatment of the tooth involved. Thereafter, in a period of eleven days, a significant increase of the lesion could be observed, resulting in visible facial asymmetry. The occlusal radiographic view showed an area of bone destruction and abnormal bone formation in the region. The external cortical portion showed clear radiopacity resembling sunrays, suggesting the diagnosis of osteosarcoma. The treatment comprised partial mandibulectomy and reconstruction of the area, using bone of the rib and skin graft from the buttock for the oral mucosa involved. Eight months after surgery, there was local recurrence of the lesion and the patient died approximately one year after relapse.

## INTRODUCTION

Osteosarcoma (OS) is the most common primary malign bone neoplasm, predominantly occurring in long bones and occasionally in the maxillofacial area^1^. Approximately 5% of OS start in maxillary bones and the mandible is the most involved site^2^. Osteosarcoma of the maxilla usually involves adults with ages ranging between the third and fourth decades of life. In addition, metastases are rare and the prognosis is significantly better when compared to its counterpart in long bones^3^. The World Health Organization (WHO) lists several variants that differ in location, clinical behavior and level of cellular atypia. The conventional or classical osteosarcoma is the most frequent variant, which develops in the medullary region of the bone and can be subdivided in osteoblastic and chondroblastic histological types, depending on the type of extracellular matrix produced by tumor cells^4^.

This paper presents a case of osteosarcoma of the mandible that was first diagnosed and treated as a dental periapical lesion and aims at comparing its clinical and microscopic findings with other similar cases that have been previously published in the literature.

## LITERATURE REVIEW

The term osteosarcoma refers to a heterogeneous group of malignant neoplasias that affect the formation of bones or mesenchymal tissue, with histopathological evidence of osteogenic differentiation.

Chindia et al. (1998)^5^ reported 14 cases of osteosarcomas of the maxillary bones, being 11 in the maxilla, 2 in the mandible and 1 in the zygomatic arch. Patients' ages ranged from one week to 50 years old (mean age of 29.7 years), equally distributed between genders. The most common clinical aspects were pain and fast volume increase, while the radiographic and histological aspects were considerably diverse. The authors reported that at least 6 of the patients who were followed up from 2 to 6 months had extensive recurrences that led to death. Treatment approaches were chemotherapy, radiotherapy and surgery, isolated or in combination.

A retrospective review of clinical, radiographic and histopathological data of 25 patients was conducted with the purpose of comparing the clinical behavior of tumors and analyzing the differences reported for tumors in other sites^4^. Mean age at presentation of primary lesions was 36.9 years (10-87 years), with slight prevalence of females. The most common aspects of presentation were: tumor volume increase, pain, ulceration and neurological disorder; radiographic aspects showed radiopaque and radiolucent areas. Histologically, there was immature bone trabeculae, which was separated by a stroma that cytologically ranged from low to high grade. In some areas, marked atypia and mitotic activity could be observed. Most lesions had areas of chondroid formation, in addition to neoplastic osteoid formation. The main complication was local recurrence. Metastases were rare and occurred isolated or at a late stage in disease progression.

Mardinger et al. (2001)^6^ presented 14 cases of OS of the maxilla and conducted their discussion based on a literature review of 774 cases reported in the English literature. Differences between OS of the maxilla and OS of long bones were also analyzed. Patients' ages ranged from 8 to 78 years (mean age of 33 years). Epidemiological data was reviewed, as well as treatment modalities and survival. Out of the 14 patients, 6 (42%) had tumor in the mandible, while 8 (58%) had it in the maxilla. The histological types found were: chondroblastic, osteoblastic, fibroblastic and one similar to malignant fibrous histiocytoma. High pathological grade (3 or 4) was detected in 13 cases, while only one mandibular case had low pathological grade (1). All patients underwent surgical resection and immediate reconstruction. Adjuvant therapy included postoperative radiation and post- and preoperative chemotherapy.

Takahama Junior et al. (2003)^7^ have recently evaluated the clinical and pathological aspects and the immunohistochemical expression of p53, MDM2, PCNA, and KI67 proteins in 25 cases of OS of the head and neck. The mean age of patients was 29 years and the most common site was the mandible (60%). The predominant histological type was chondroblastic (72%). The immunohistochemical analysis was positive in 52% of cases for p53, 24% for MDM2, 84% for CDK4, 92% for PCNA and 88% for KI67. Most patients were treated with surgery alone or in association with chemotherapy. Five-year and 10-year survival rates were 59% and 49%, respectively, and the most important prognostic factors were previous exposure to radiation and osteoblastic histological type.

## CASE REPORT

**Figure 1:**
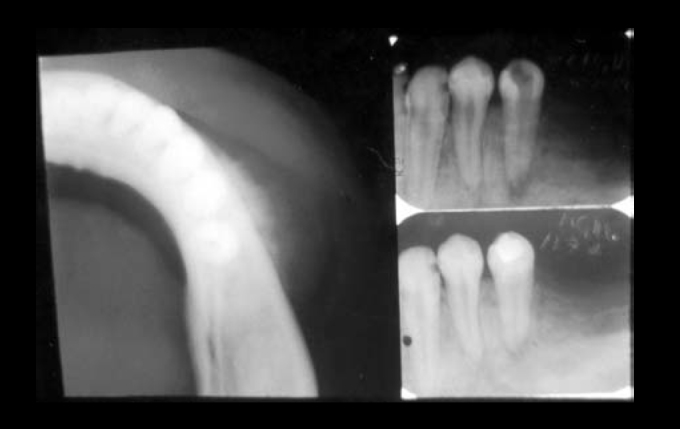
Occlusal radiographic view showing the external cortical portion with radiopacity similar to “sunrays” (on the left). Periapical X-rays showing increased periapical space of the premolar tooth (on the right).

The 20-year-old female patient was referred to the Oral Pathology area of the UFRN Odontology department for examination of a volume increase in the lower left premolar region (PM). The patient had previously attended an odontological center reporting a slight volume increase in the described area. It had hard consistency, painless upon palpation, with no other manifestations. The periapical radiography suggested radicular reabsorption and diffuse bone rarefaction of the 34 periapex; the patient was followed up by endodontic treatment of the tooth with necrotic pulp (Figure 1). After 11 days, the patient returned to the clinic reporting no improvement. It could be noticed that there was fast progression of the disease, with clear facial asymmetry, associated with numbness on the left side of the lower lip, reported by the patient. Upon palpation, there was no involvement of the lymphatic chain in the cervicofacial area and, in the intraoral examination, it could be noticed that the vestibular region of the lower left PM was swollen, red and bleeding. The lesion was growing fast, but there was no change in the general physical status of the patient. A complete blood test and urine test were ordered, and their results were normal. A radiographic exam was also ordered, encompassing the following radiographic views: lateral oblique view of the left mandible, occlusal and periapical views, and posteroanterior view of the chest to look for possible pulmonary metastasis. The occlusal radiography image showed bone destruction and abnormal formation in the region, with ground glass appearance, masking details in the bone trabeculae. The external cortical view showed significant radiopacity similar to “sunrays”, suggesting a diagnosis of osteosarcoma (Figure 1). The patient was then referred to the UFRN oral diagnostic service, and later underwent a biopsy in the laboratory of Clinical Pathology and Surgery of the city of Natal, where a histopathological assessment confirmed the clinical suspicion of osteosarcoma.

## DISCUSSION

Primary osteosarcomas represent a heterogeneous group of malignant bone tumors, characterized by the diversity of histological aspects and clinical and biological behavior. They occur more frequently in long bones and rarely in the maxilla^4^.

OS of gnathic bones usually involves adults with ages ranging between the third and fourth decades of life^3^, ^4^, ^6^, ^8^. In this paper, we report an OS case involving a 20-year-old female. According to Slootweg and Muller (1985)^7^, age can be an important factor in the differentiation of OS in several anatomical regions and in prognostic estimates. For those authors, older patients have better prognosis due to an increased resistance to the tumor.

Some preexisting etiologic conditions can lead to the development of OS, such as previous exposure to radiation, fibrous dysplasia, Paget's bone disease and local trauma. This may suggest an association between this neoplasm and excessive cellular activity^6^. In the case reported in that study, there was no involvement of any of the predisposition conditions.

**Figure 2:**
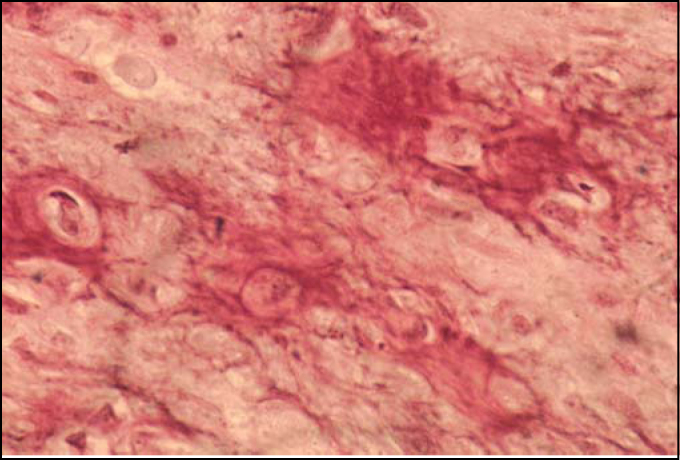
Photomicrography showing osteoid area and mild cellular pleomorphism (HE/400x).

**Figure 3:**
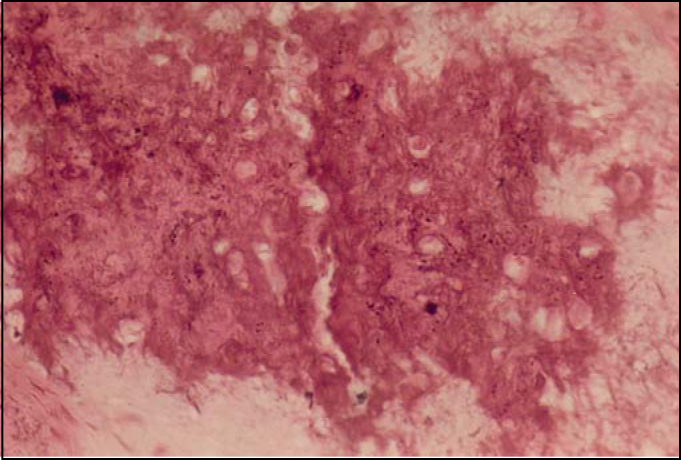
Photomicrography showing extensive area of osteoid deposition (HE/200X).

In the maxillomandibular region, most osteosarcomas have an osteoblastic nature, with deposition of a variable amount of osteoid matrix, with minimal cytological atypia and usually with well-differentiated lesions^4^, ^5^. In our case, histologically the tumor was composed of cells with shapes varying between oval and spindle-like with mild cellular pleomorphism, responsible for the deposition of extensive osteoid areas, typical of the osteoblastic type (Figures 2 and 3). Takahama Junior et al. (2003)^7^, in a study with 25 cases of OS, observed that, according to the histological type of the tumor, patients with the chondroblastic type had a higher survival rate when compared to patients with the osteoblastic type (p = 0.02).

There are differences in the clinical behavior of tumors in maxillary bones that have a strong influence on disease progression, treatment and outcome^6^. Osteosarcomas of maxillary bones are less aggressive than those of long bones, since they rarely generate metastasis and are present in slightly older age groups. In addition, early diagnosis is favored by aesthetical and functional reasons, especially in the maxillofacial region^8^.

In their literature review, Carnelio et al. (2002)^2^ stated that the 5-year survival rate for primary osteosarcomas of maxillary bones ranges from 30 to 40%. A survival rate over 80% was reported for patients that underwent early radical resection.

**Figure 4:**
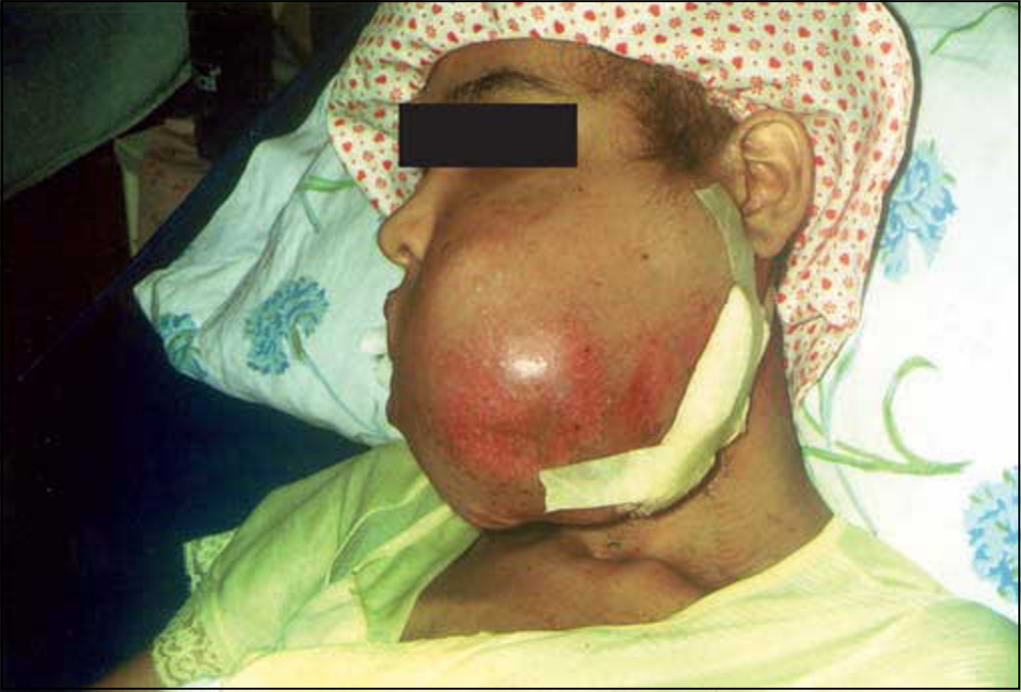
Extraoral clinical aspect of recurring tumor 8 months after surgery.

The case reported in this paper had survival lower than what is observed in most OS of gnathic bones reported in the literature. It has shown to be biologically similar to its counterpart in long bones in terms of aggressiveness, since there was local recurrence of the lesion eight months after surgery (Figure 4), leading to the patient's death one year after relapse. Therefore, the patient's survival was less than two years. A low survival rate was also observed by Takahama Junior et al. (2003)^7^ in all the patients enrolled in the study who developed local recurrence, since they died due to the disease within less that 5 years of follow-up.

Osteosarcoma treatment is well established in long bones, but it is not well understood when the condition involves the mandible or maxilla. It is clear that chemotherapy is beneficial in OS of long bones, leading to significant changes in disease-free survival rate (from 20% in the 1960's to 70% in the 1980's). This improvement did not include OS of the maxilla, due to their rare occurrence and to lack of standardized chemotherapy protocols, which makes it difficult to evaluate the efficiency of adjuvant therapy^6^.

In most cases, the therapy of choice is radical surgical excision, since it provides a 5-year survival rate over 80%. As to chemotherapy, it seems that it does not have much impact in the survival rates of patients with OS of the maxilla. This can be explained by the fact that metastases are rare and late, occurring in only 18% of cases, and that local recurrence of the lesion is still the leading cause of death^6^.

In the case presented in our study, the lesion was diagnosed early, surgical treatment was provided with hemimandibulectomy associated with chemotherapy, which were not effective in avoiding relapse, showing the intrinsic aggressiveness of the tumor.

## CLOSING REMARKS

More accurate definitions of the biological behavior of OS in maxillary bones are required to establish an effective therapeutic regimen in order to increase the survival rate of patients. Considering how rare this disease type is and particularly taking into account the fast progression and aggressiveness of the case reported in this paper, it is clear that the presentation of clinical cases represents a major contribution to better understanding osteosarcomas involving maxillary bones.
